# Machine learning analyses of methylation profiles uncovers tissue‐specific gene expression patterns in wheat

**DOI:** 10.1002/tpg2.20027

**Published:** 2020-06-03

**Authors:** Amidou N'Diaye, Brook Byrns, Aron T. Cory, Kirby T. Nilsen, Sean Walkowiak, Andrew Sharpe, Stephen J. Robinson, Curtis J. Pozniak

**Affiliations:** ^1^ Department of Plant Sciences and Crop Development Centre University of Saskatchewan Saskatoon SK Canada S7N 5A8; ^2^ Global Institute for Food Security Saskatoon SK Canada S7N 0W9; ^3^ Saskatoon Research and Development Centre Agriculture and Agri‐Food Canada Saskatoon SK Canada S7N 0X2

## Abstract

DNA methylation is a mechanism of epigenetic modification in eukaryotic organisms. Generally, methylation within genes promoter inhibits regulatory protein binding and represses transcription, whereas gene body methylation is associated with actively transcribed genes. However, it remains unclear whether there is interaction between methylation levels across genic regions and which site has the biggest impact on gene regulation. We investigated and used the methylation patterns of the bread wheat cultivar Chinese Spring to uncover differentially expressed genes (DEGs) between roots and leaves, using six machine learning algorithms and a deep neural network. As anticipated, genes with higher expression in leaves were mainly involved in photosynthesis and pigment biosynthesis processes whereas genes that were not differentially expressed between roots and leaves were involved in protein processes and membrane structures. Methylation occurred preponderantly (60%) in the CG context, whereas 35 and 5% of methylation occurred in CHG and CHH contexts, respectively. Methylation levels were highly correlated (r = 0.7 to 0.9) between all genic regions, except within the promoter (r = 0.4 to 0.5). Machine learning models gave a high (0.81) prediction accuracy of DEGs. There was a strong correlation (p‐value = 9.20×10^−10^) between all features and gene expression, suggesting that methylation across all genic regions contribute to gene regulation. However, the methylation of the promoter, the CDS and the exon in CG context was the most impactful. Our study provides more insights into the interplay between DNA methylation and gene expression and paves the way for identifying tissue‐specific genes using methylation profiles.

**Abbreviations**
CART: classification and regression trees
DEG: differentially expressed gene
DMR: differentially methylated region
GO: gene ontology
KNN: K‐nearest neighbors
LASSO: least absolute shrinkage and selection operator
LDA: linear discriminant analysis
LR: logistic regression
ML: machine learning
NB: Naïve Bayes
RFE: recursive feature elimination
SVM: support vector machine
WGBS: whole‐genome bisulfite sequencing

## 1 INTRODUCTION

DNA methylation is a major mechanism of epigenetic modification in eukaryotic organisms. High‐throughput sequencing technologies combined with whole‐genome bisulfite sequencing (WGBS) have allowed the decoding of genome‐wide methylation at the nucleotide level for plants (Hardcastle, 2013; Rusk, 2008; Yong, Hsu, & Chen, 2016), animals and humans (Doherty & Couldrey, 2014; Shiratori et al., 2016). Recently, WGBS has been widely applied to profiling the DNA methylation in many plant species, including *Arabidopsis* (Cokus et al., 2008), rice (He et al., 2010; Liu et al., 2017) and soybean (Song et al., 2013). In wheat, DNA methylation studies have been conducted for genome‐wide profiling (Gardiner et al., 2015) or the dynamics of methylation in diverse environmental stresses (Geng et al., 2019; Kaur, Grewal, & Sharma, 2018; Kumar, Beena, Awana, & Singh, 2017.

Plants have developed various mechanisms to adapt to daily environmental conditions, and the regulation of gene expression through both transcriptional regulation and post‐transcriptional regulation is very important to their survival. In both animals and plants, DNA methylation status may affect gene expression, splicing, and polyadenylation (Huettel et al., 2006; Regulski et al., 2013; Tsuchiya & Eulgem, 2013), and can influence DNA repair, recombination and meiotic crossover in euchromatic regions (Dowen et al., 2012; Melamed‐Bessudo & Levy, 2012; Mirouze et al., 2012). DNA methylation is a dynamic regulator of the plant genome during development and environmental adaptions (Bartels et al., 2018; Bräutigam & Cronk, 2018; Flores, Wolschin, & Amdam, 2013; Thiebaut, Hemerly, & Ferreira, 2019). For example, DNA methylation plays an important role in floral symmetry (Cubas, Vincent, & Coen, 1999), flowering time (Soppe et al., 2000), fruit ripening (Manning et al., 2006), parental imprinting (Huh, Bauer, Hsieh, & Fischer, 2008), sex determination (Martin et al., 2009), stomatal development (Tricker, Gibbings, López, C.M., & Wilkinson, 2012; Yamamuro et al., 2014), pigmentation (Li et al., 2018) and plant immunity (Geng et al., 2019; Jeon et al., 2015). The methylation levels and patterns vary across species, tissues or organs, developmental status and the biotic and abiotic environment (Wibowo et al., 2018; Zhang, Lang, & Zhu, 2018b). Therefore, variation in DNA‐methylation may contribute to phenotypic plasticity in plants.

In plants, methylation of DNA generally occurs at cytosine bases and is observed in three different sequence contexts that differ in the adjacent nucleotide residues: CG, CHG and CHH contexts (H = A, T, or C), which are maintained by individual enzymatic pathways (Chan, Henderson, & Jacobsen, 2005; Henderson & Jacobsen, 2007; Law & Jacobsen, 2010). Methylation at the CG sequence context is the major type of cytosine methylation, as it is widely distributed not only in heterochromatic, such as TEs and repeat sequences, but also in euchromatic regions of genes (Wang et al., 2010; Zhang, Kimatu, Xu, & Liu, 2010). The mechanisms responsible for the establishment and maintenance of cytosine methylation have been best studied in *Arabidopsis* (Chan et al., 2005; Gehring & Henikoff, 2008; Stroud et al., 2013; Vongs, Kakutani, Martienssen, & Richards, 1993), where *de novo* methylation is catalyzed by Domains‐Rearranged Methyltransferases2 (DRM2). After methylation occurs, it is maintained by different enzymes depending on the context; CG by DNA Methyltransferase1 (MET1), and CHG by Chromomethylase3 (CMT3), and CHH methylation is not maintained (Chan et al., 2005; Law & Jacobsen, 2010).

The primary function of DNA methylation is the maintenance of genome stability (Meng et al., 2015; Zhou & Robertson, 2016) but the functional role of DNA methylation in gene expression is complex and linked to the location of the methylated cytosine across the gene (Brenet et al., 2011; Flores et al., 2013; Jones, 2012). Several studies have shown that promoter methylation represses gene expression while gene body methylation is usually associated with actively transcribed genes (Ball et al., 2009; Hellman & Chess, 2007; Li et al., 2012; Spainhour, Lim, Yi, & Qiu, 2019). However, others have found that methylation of promoter regions is positively correlated with gene expression (Xu et al., 2018) and that DNA methylation across gene body has limited contribution to gene expression (Meng et al., 2016). These observations suggest a complex interplay of DNA methylation with gene expression (Yu et al., 2013). While some published results have shown the tissue/organ specificity of methylation signatures (Bhatia, Khemka, Jain, & Garg, 2018a; Wibowo et al., 2018) leading to heritable phenotypic variations, others have demonstrated tissue‐specific gene regulation (Huang, Zheng, Yuan, & McGinnis, 2018; Sonawane et al., 2017; Zhang et al., 2017). However, it is still unclear whether methylation profiles can help identifying tissue‐specific genes and therefore, potential candidate genes for specific traits or biological functions.

**Core Ideas**

- There is a very complex relationship of DNA methylation with gene expression.
- It is still unclear whether there is interaction between methylation levels across genic regions and which site has the biggest impact on gene regulation.
- Our study highlights the potential of machine learning for accurately (accuracy = 0.81) identifying tissue‐specific gene regulation patterns, using methylation profiles.

With the increasing availability of ‘big data’ and high‐performance computing, machine learning (ML) is being deployed in various scientific fields, including robotics (Takahashi, Kim, Ogata, & Sugano, 2017), bioinformatics (Olson, Cava, Mustahsan, Varik, & Moore, 2018), biochemistry (Costello & Martin, 2018), medical diagnosis (Doan & Carpenter, 2019; Jurmeister et al., 2019), meteorology (Rhee & Im, 2017) and climatology (Fang, Shen, Kifer, & Yang, 2017). In agriculture, ML algorithms have been successfully applied to predict yield in many crops (see Mishra, Mishra, & Santra, 2016 for a review), including wheat (Jeong et al., 2016; Pantazi, Moshou, Alexandridis, Whetton, & Mouazen, 2016) and maize (Aghighi, Azadbakht, Ashourloo, Shahrabi, & Radiom, 2018). Because ML algorithms can potentially approximate any function, ML may easily uncover genuine patterns within complex datasets (Xu & Jackson, 2019; Zitnik et al., 2019).

This study aimed to investigate the potential of cytosine methylation patterns for identifying differentially expressed genes between roots and leaves, using machine learning and artificial neural networks.

## 2 MATERIALS AND METHODS

### 2.1 Plant material

The allohexaploid wheat (*Triticum aestivum* L.) cultivar Chinese Spring was grown in Cyg growth pouches (Mega International, USA) supplemented with 50% Hoagland's solution in a growth chamber providing a constant temperature of 20°C with a 12‐h photoperiod for 14 days. Leaf and root tissues from plants that had developed to the three‐leaf stage (Zadok's stage 13) were harvested for RNA and DNA extraction.

### 2.2 RNA extraction

Total RNA was prepared by grinding sampled tissues in liquid nitrogen and processed using the Qiagen RNeasy Plant Mini Kit (Qiagen, USA) following the manufacturer's instructions. RNA samples were treated with DNAse I to ensure there was no contaminating DNA. RNA integrity was evaluated using a Bioanalyser RNA 6000 nano chip (Agilent, Santa Clara, CA), and was quantified using a Qubit Broad Range (BR) assay kit (Thermofisher, USA).

### 2.3 cDNA library construction and sequencing

Individually barcoded cDNA libraries were prepared using the Truseq v2 unstranded kit (Illumina) following the manufacturer's instructions. Library integrity was checked using a Bioanalyser High Sensitivity DNA analysis kit (Agilent, Santa Clara, CA) and each library was quantified using a Qubit High Sensitivity (HS) DNA assay kit (Thermofisher, USA). Libraries were diluted to 10 ng µl^−1^, and paired‐end sequence reads were generated using either the Illumina HiSeq2500 (100 bp) or the HiSeq4000 (150 bp) platform. Sequencing was performed at the McGill University and Génome Québec Innovation Centre, Montréal, Canada.

RNAseq alignments were performed using STAR v 2.7 (Dobin et al., 2012), using the Chinese Spring genome RefSeqv1.0 as the reference (Appels et al., 2018), with default parameters except for –outFilterMultimapNmax 10, –outFilterMismatchNoverLmax 0.02, –alignIntronMin 10, –alignIntronMax 12000, –outFilterMultimapScoreRange 0 and –outFilterMatchNminOverLread 0.9.Stringtie (Pertea et al., 2015) was used to assemble transcripts and these data were merged with the Chinese Spring high confidence gene models. A transcript count matrix was generated using Salmon algorithm (Patro, Duggal, Love, Irizarry, & Kingsford, 2017) and was subsequently fed into the DESeq2 R package (Love, Huber, & Anders, 2014). A normalized count matrix was produced using the VST function of DESeq2.

### 2.4 Nuclear DNA purification

The same root and leaf tissues used for RNA sequencing were also used for DNA isolation. To selectively enrich for nuclear DNA, nuclei were extracted using the protocol described by Zhang, Zhao, Ding, Paterson, and Wing (1995) with the following modifications. Intact nuclei were visually inspected and their concentration estimated using a haemocytometer. Enriched nuclei samples were lysed and high‐quality DNA was purified using proteinase K and phenol chloroform extraction followed by ethanol precipitation. Purified DNA was quantified using a Qubit high sensitivity DNA kit (Thermofisher, USA). A total of 500 ng of nuclear DNA was spiked with 270 pg of Lambda DNA and unmethylated cytosine bases were converted to uracil by sodium bisulfite treatment using the EZ DNA Methylation‐GoldTM Kit (Zymo research corp, Irvine, CA) as per the manufacturer's instructions.

### 2.5 Whole genome bisulfite sequencing (WGBS) and sequence processing

A total of 500 ng of DNA from leaf or root tissues were used as input for the generation of whole genome bisulfite sequencing libraries using the Truseq DNA methylation kit, (Illumina, Madison, WI). An average insert size of 240 bp was selected for library construction using AMPure XP beads (NEB, Ipswich, MA). Library size and quality were assessed using a Bioanalyzer High Sensitivity DNA chip (Agilent, Santa Clara, CA) and further quantified using a Qubit 2.0 high sensitivity dsDNA kit (Thermofisher, USA) prior to library construction. Five independent libraries were constructed for each tissue. Paired‐end sequence data (125 bp) from bisulfite converted DNA was generated using the Illumina HiSeq 2500 v4 platform (Genome Quebec, Montreal, Canada).

Sequencing reads were processed to remove low quality base calls and adaptor sequences using Trim galore (Krueger, Kreck, Franke, & Andrews, 2012). The base conversion efficiency obtained from the sodium bisulfite treatment was estimated by aligning sequenced reads to the Lambda genome. Since cytosine methylation is absent in Lambda, no methyl group should protect cytosine bases from conversion to uracil. Bisulfite conversion efficiency in excess of 98% was observed. The remaining high‐quality paired‐end sequencing reads were aligned to the assembled Chinese Spring genome sequence RefSeqv1.0 using Bismark version 0.16.1 (Krueger & Andrews, 2011) in conjunction with Bowtie2, version 2‐2.1.0, using the options ‐N 0 ‐D 15. After alignment, duplicate reads were removed using the ‘deduplicate_bismark_alignment.pl’ Perl script. The overall wheat methylome was determined by combining counts at each cytosine across each of the sequenced leaf libraries. Cytosine residues with insufficient read depth and ambiguous methylation calls were excluded from further analysis. All sequence data for seedlings are already available (Appels et al., 2018), and data for roots has been deposited to the short‐read archive (https://www.ncbi.nlm.nih.gov/bioproject/PRJNA436361).

### 2.6 Identification of differentially methylated regions (DMRs)

For the genome‐wide methylation profile, the methylation ratio of each site was computed as

$$\beta -\mathrm{value}=C_{i}/\left(C_{i}+T_{i}\right)$$

with C = number of reads supporting methylated cytosine, T = number of reads supporting unmethylated cytosine and i = position of the cytosine.

At the gene level, we first identified genic regions (promoter, CDS, exon, intron and downstream) based on the annotations of Chinese Spring genome (Appels et al., 2018). Then, we computed the weighted methylation levels (Schultz, Schmitz, & Ecker, 2012) for each feature as follow:

$$\sum_{\left(i=1\right)}^{n}\mathrm{Ci}=\sum_{\left(i=1\right)}^{n}\left(\mathrm{Ci}+\mathrm{Ti}\right)$$

with i = genic region and n = total number of cytosine positions.

### 2.7 Identification of differentially expressed genes (DEGs) between roots and leaves

Differential expression analysis between roots and leaves was performed using DESeq2 that provides statistical metrics (p‐value, adjusted p‐value and log2 fold change), based on a negative binomial distribution (Anders & Huber, 2010). Three gene expression classes were generated with regards to roots as followed:

‘Downregulated’ when adjusted p‐value ≤ .05 and log2 fold change ≤ −2
‘Upregulated’ when adjusted p‐value ≤ .05 and log2 fold change ≥ 2
‘Not differentially expressed’ when adjusted p‐value > .05.

### 2.8 Gene ontology (GO) enrichment analysis of differentially expressed genes

To search for patterns of biological processes, molecular functions and cellular components among the three gene expression classes (downregulated, upregulated and not differentially expressed), the Gene Ontology (GO) terms (Ashburner et al., 2000) were hierarchically investigated using the ClusterProfiler R package (Yu, Wang, Han, & He, 2012).

### 2.9 Prediction of gene expression classes

Prior to building predictive models, the contribution of individual gene features was evaluated using two techniques:

1. The Recursive Feature Elimination (RFE) implemented in Scikit‐learn python module (Pedregosa et al., 2011); the goal of RFE is to select features by recursively removing the features of least importance. First, the estimator is trained on the initial set of features and the importance of each feature is estimated. Then, the least important features are pruned from current set of features. That procedure is recursively repeated on the pruned set until there is no more feature to remove.
2. The Least Absolute Shrinkage and Selection Operator (LASSO) model that penalizes the absolute size of the regression coefficients, based on the value of a tuning parameter λ. As a result, the LASSO shrinks the coefficients of irrelevant variables to zero, thus performing automatic variable selection (Tibshirani, 1996).

Six machine learning (ML) algorithms implemented in Scikit‐learn were used to predict genes expression classes between roots and leaves, using the methylation patterns as features:

1. Naïve Bayes (NB); Naïve Bayes is a probabilistic machine learning model based on the Bayes theorem. For our classification problem, The Bayes theorem can be rewritten as
$$P\left(y|X\right)\;=\frac{P\left(X|y\right)P\left(y\right)}{P\left(X\right)}\;$$

where P denotes the probability, y represents the class variable given the conditions and X being the features.

1. Logistic regression (LR); LR is a classification algorithm used to assign observations to a discrete set of classes, using a sigmoid function that returns a probability value which can then be mapped to two or more discrete classes. Instead of fitting a straight line or hyperplane, the logistic regression model uses the logistic function to squeeze the output of a linear equation between 0 and 1.
2. Linear discriminant analysis (LDA); the particularity of LDA is that it models the distribution of predictors separately in each of the response classes, and then it uses Bayes’ theorem to estimate the probability.
3. K‐nearest neighbors (KNN); KNN is a non‐parametric method used for both classification and regression problems. For classification, an object is classified by a majority vote of its neighbors, with the object being assigned to the class most common among its k nearest neighbors.
4. Classification and regression trees (CART) (Breiman, Friedman, Olshen, & Stone, 1984); CART is a decision tree algorithm for both classification and regression. It is a recursive algorithm, which partitions the training data set by doing binary splits. In their simplest form, decision tree algorithms are hierarchical if‐else statements that can be applied to predict a result based upon data. The if‐else statements are chosen to maximize a notion of information gain and reduce the variability in the underlying (two) children nodes. In contrast with general tree‐based methods that may allow multiple child nodes, CART always creates a binary tree. A large tree is first generated, then pruned to a size that has the lowest cross‐validation estimate of error (Loh, 2014).
5. Support vector machine (SVM) (Vapnik, 1995); SVM uses a nonlinear mapping function to map samples from the predictor space to a high‐dimensional feature space and perform linear regression in the latter space (Witten & Frank, 2005).

In an attempt to improve the prediction accuracy, we built an Ensemble model combining all six ML algorithms, using the ‘VotingClassifier’ module from Scikit‐learn. In this technique, each model is used to make predictions for each data point. The predictions by each model is considered as a ‘vote’. The predictions that come from the majority of the models are used as the final prediction. Finally, we built a fully connected deep neural networks in Keras (https://keras.io/) coupled with TensorFlow (https://www.tensorflow.org/), using the following design: two hidden layers of 100 and 40 neurons, with the Rectified Linear Unit ‘relu’ as activation function; for compiling the model, we used the ‘categorical_crossentropy’ loss function, the ‘adam’ optimizer and 200 epochs (iterations). Deep neural networks belong to the class of representation learning models that can automatically find the underlying representation of data without handcrafted input of features. Deep learning discovers intricate structure in large data sets by using the backpropagation algorithm to indicate how a machine should change its internal parameters that are used to compute the representation in each layer from the representation in the previous layer (LeCun, Bengio, & Hinton, 2015). Thus, deep learning can approximate almost any function, although it may be very challenging to find the right parameters (Hornik, Stinchcombe, & White, 1990). To account for the highly unbalanced structure of the data, we used the over‐sampling minority class technique (Barua et al., 2011; Dubey, Zhou, Wang, Thompson, & Ye, 2014) for all of the predictive models.

## 3 RESULTS

### 3.1 Genome‐wide methylation patterns

**FIGURE 1:**
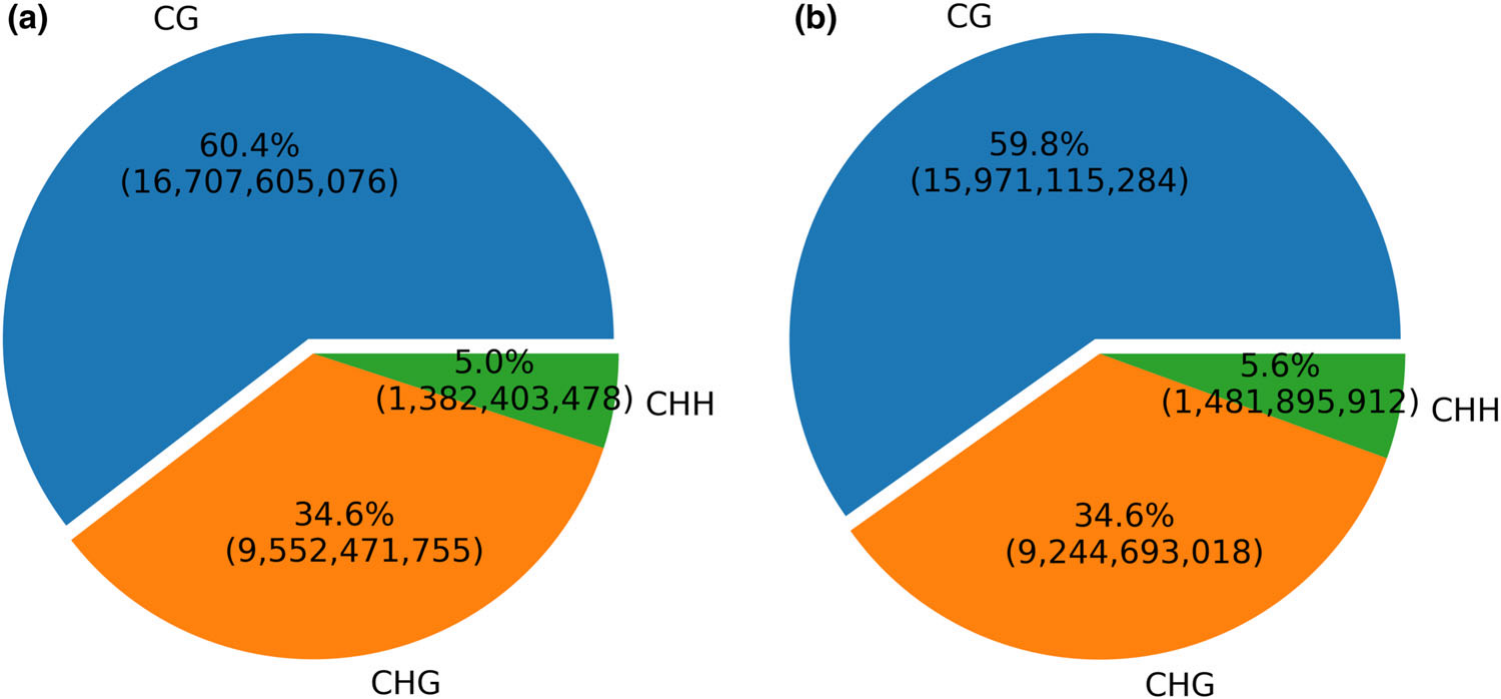
Proportion of methylated cytosines in CG (blue), CHG (orange) and CHH (green) contexts in roots (A) and leaves (B)

In roots, from the 27,642,479,588 methylated cytosine residues across the genome, 60.4, 34.6 and 5.0% occurred in CG, CHG and CHH contexts, respectively (Figure 1a). These proportions were similar among chromosomes (Supplemental Figure S1A). Similarly, a total of 26,697,705,209 cytosine residues were methylated in leaves, of which 59.8, 34.6 and 5.6% occurred in CG, CHG and CHH contexts, respectively (Figure 1b). The same patterns were observed among chromosomes (Supplemental Figure S1B).

**FIGURE 2:**
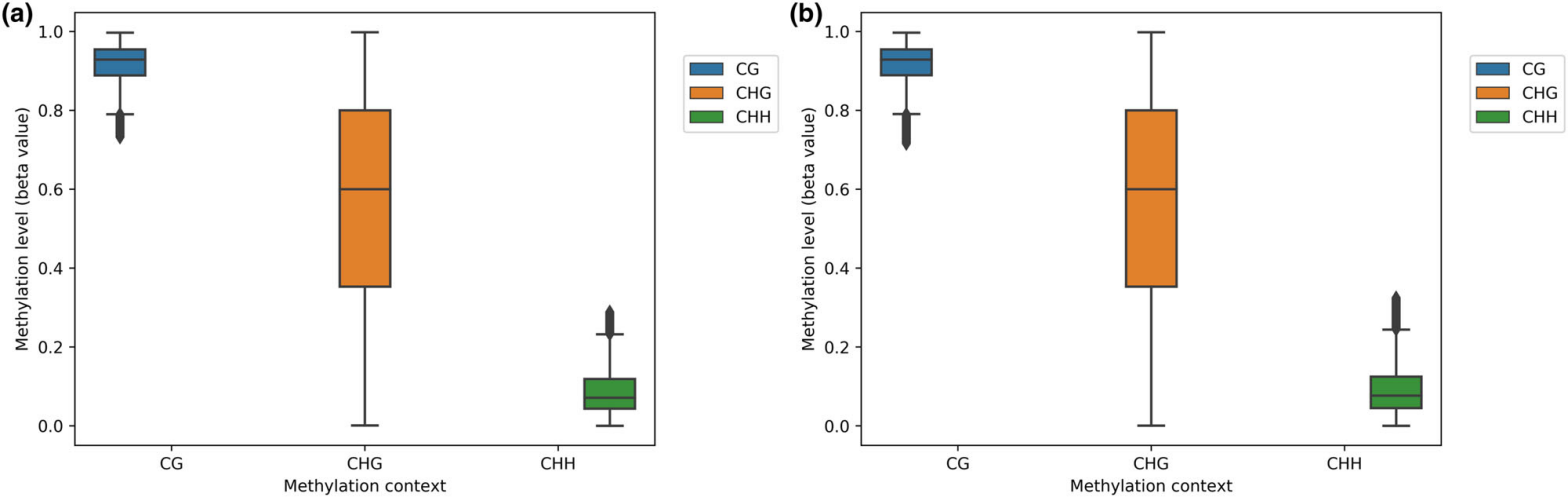
Methylation levels in CG (blue), CHG (orange) and CHH (green) contexts across the genome in roots (A) and leaves (B)

There was no difference in DNA methylation patterns between chromosome strands for both roots (Supplemental Figure S2A) and leaves (Supplemental Figure S2B); therefore, methylation data of both chromosome strands were combined for the subsequent analyses. However, the proportion of methylcytosines depends on the contexts in which the methylation occurs. On average, the methylation level was the highest (beta‐value = 0.91) in CG context while very low (beta‐value = 0.1) in CHH context for both roots (Figure 2a) and leaves (Figure 2b). For CHG context, the average methylation ratio was 0.57, with a relatively wide range of variation (minimum = 0, maximum = 0.99).

### 3.2 Methylation profiles at the gene level

The genomic locations of methylated cytosine residues were assessed using the annotated genome of Chinese Spring. As several annotated genes lacked a defined 3′‐UTR and/or 5′‐UTR region, these attributes were removed from the analyses. As a result, DNA methylation sites covered 100,028 genes of which 76.8% were complete genes having all of the attributes (promoter, CDS, exon, intron and downstream region), whereas 15.9% (15,905) were single exon genes. To take into account single exon genes in the predictive analyses, the intron was removed from the attributes of the complete genes as a feature should not contain any missing value for training machine learning algorithms. Nonetheless, prior to excluding intron from the features, the contribution of intronic methylation levels to the predictive models was evaluated using LASSO algorithm (Supplemental Figure S3). The methylation in intronic regions showed only little contribution to the models. Therefore, the final matrix of features included the promoter, CDS, exon, downstream region and the three methylation contexts (CG, CHG and CHH).

**FIGURE 3:**
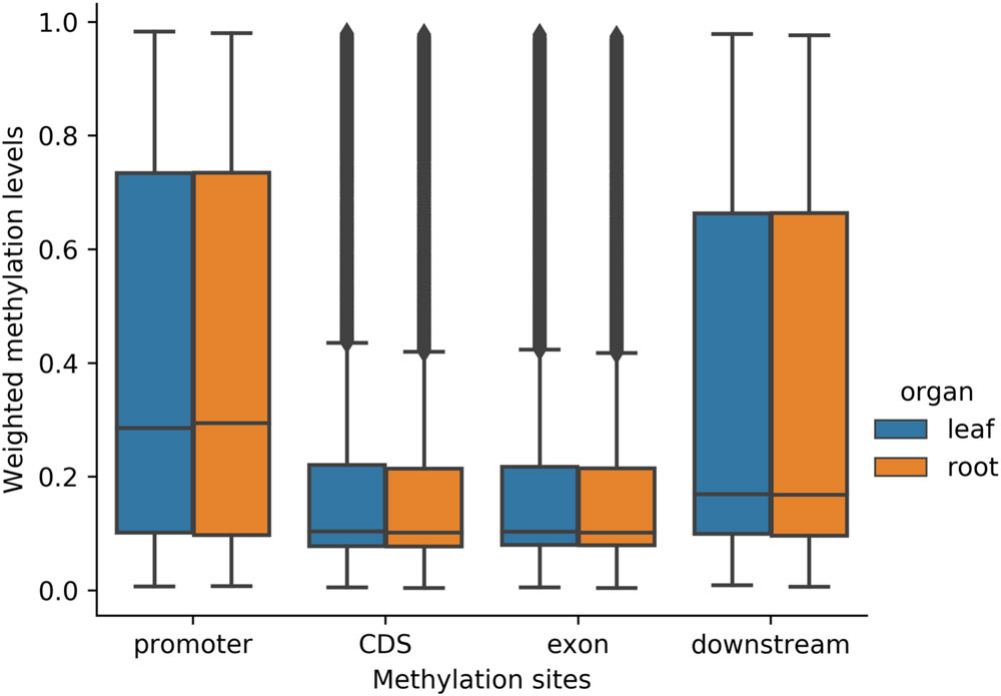
Comparison of methylation levels between roots and leaves across genic regions

The methylation patterns were similar between root and leaf for each genic region (Figure 3). However, the methylation levels were significantly different (p‐value < .05) between some, but not all of the genic regions (Table 1). For example, the promoter consistently showed the highest methylation ratio in each context (0.91, 0.45 and 0.10 in CG, CHG and CHH, respectively), whereas there was no difference of methylation levels between CDS and exons in each methylation context.

**TABLE 1:** Comparison of methylation levels between genic regions in each methylation context. Means in the same row that share a common letter are not significantly different (p‐value > .05)

Context	Promoter	CDS	Exon	Downstream
CG (n = 145,561)	0.91a (±0.04)	0.88c (±0.04)	0.88c (±0.04)	0.89b (±0.05)
CHG (n = 239,544)	0.45a (±0.22)	0.14d (±0.12)	0.14d (±0.12)	0.35b (±0.21)
CHH (n = 245,523)	0.10a (±0.03)	0.09b (±0.03)	0.09b (±0.03)	0.10a (±0.03)

### 3.3 Gene expression analyses

Prior to investigating differentially expressed genes between root and leaf, the global gene expression profiles were evaluated in each tissue, separately (Supplemental Figure S4). In both root and leaf, the distributions of gene expression were markedly right‐skewed. Because of these L‐shape distributions, any regression model for a linear correlation between methylation profiles and gene expression would result in poor performance since the slope of the curves would tend to zero or infinite.

**FIGURE 4:**
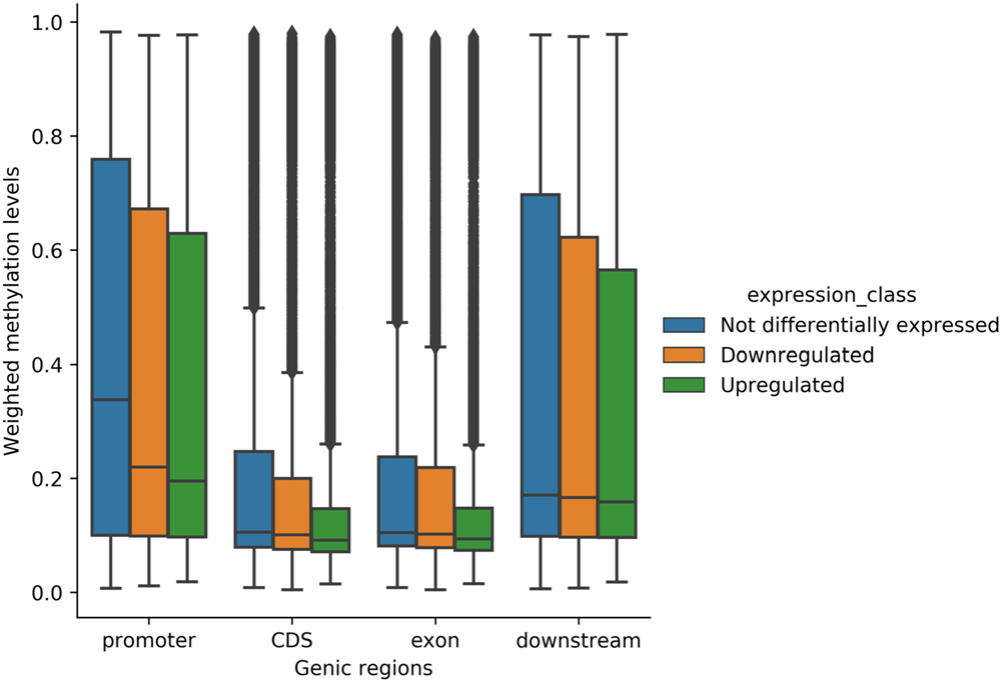
Comparison of methylation levels across genic regions between gene expression classes: downregulated in roots (orange), not differentially expressed between roots and leaves (blue) and upregulated in roots (green)

The genes harboring methylated cytosine residues were clustered into three gene expression classes (‘downregulated’, ‘upregulated’ and ‘not differentially expressed’), based on the adjusted p‐values and the log_2_ fold changes. The majority (70.44%) of the genes were not differentially expressed between roots and leaves; only 18.4 and 11.2% of the genes were upregulated and downregulated in roots, respectively. The average methylation levels were significantly (p‐value < .05) different between genes expression classes in each genic region (Figure 4). On average, the promoter showed the highest methylation level among features in each genes expression class, 0.43 (±0.33), 0.39 (±0.32) and 0.37 (±0.32) in ‘not differentially expressed’, downregulated and upregulated, respectively (Supplemental Table S1).

The different methylated regions (promoter, CDS, exon and downstream region) showed significant (p‐value < .001) correlation with gene expression in each methylation context (Table 2). In general, there was a positive correlation between features and gene expression. However, methylation in CDS and exon were negatively correlated with gene expression in CHG context. There was significant (p‐value = 9.20×10^−10^) interaction between features and gene expression classes (Table 3).

**TABLE 2:** Pearson's correlation (r, p‐value) between gene expression and methylation levels of different genic regions

Context	Promoter	CDS	Exon	Downstream
CG	0.079	0.042	0.037	0.019
	(9.87×10^−159^)	(2.97×10^−46^)	(9.15×10^−36^)	(5.40×10^−11^)
CHG	0.133	−0.015	−0.019	0.039
	(0.001)	(2.59×10^−12^)	(3.22×10^−17^)	(1.08×10^−70^)
CHH	0.030	0.076	0.077	0.026
	(2.84×10^−43^)	(8.93×10^−268^)	(5.08×10^−273^)	(4.77×10^−33^)

**TABLE 3:** Two‐way analysis of variance (ANOVA II) of methylation levels between genic regions among gene expression classes

Source	SS	df	F	P(> F)
Gene expression	727.79	2	3410.71	0.01
Features	7723.05	3	24128.94	0.01
Gene expression × Features	39.44	6	61.62	9.20×10^−77^
Residual	226693.60	2124760		

**FIGURE 5:**
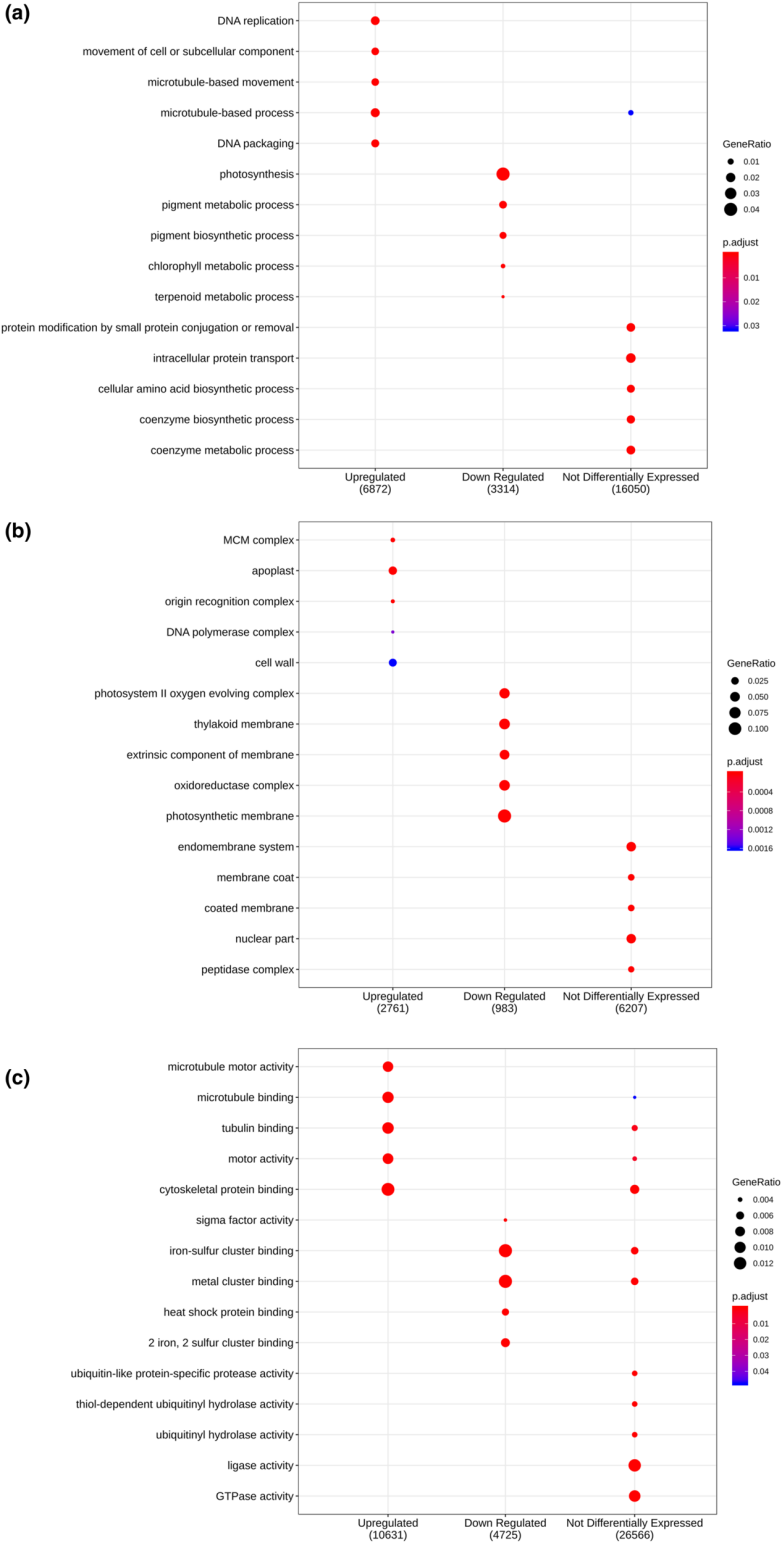
Distribution of genes between expression classes based on biological processes (A), cellular components (B) and molecular functions (C)

The GO enrichment analysis showed clear clusters of biological processes and cellular components between the three gene expression classes (Figure 5). The upregulated genes in roots were involved in five biological processes, including DNA replication, movement of cells or subcellular components, microtubule‐based movement, microtubule‐based process and DNA packaging, whereas the downregulated genes were mainly involved photosynthesis and pigment biosynthesis processes. Genes that were not differentially expressed between roots and leaves were mainly involved in protein processes (e.g., amino acid biosynthesis and protein transport). For the cellular components, the upregulated genes affected MCM complex, apoplast, origin recognition complex, DNA polymerase complex and cell wall, whereas the downregulated genes affected photosynthesis‐related components (e.g., photosynthetic membrane, photosystem II oxygen evolving complex and oxidoreductase complex). Genes where no expression difference was detected between roots and leaves were involved in membrane structures. For molecular functions (Figure 5), the upregulated genes were related to motor activities and binding functions of microtubules, tubulin and cytoskeletal protein. The downregulated genes were associated with sigma factor activity and clusters binding (iron‐sulfur, metal and heat shock protein). The genes that were not differentially expressed genes covered the whole range of molecular functions, but with a preponderance of GTPase and ligase activities.

### 3.4 Prediction of gene expression classes

**FIGURE 6:**
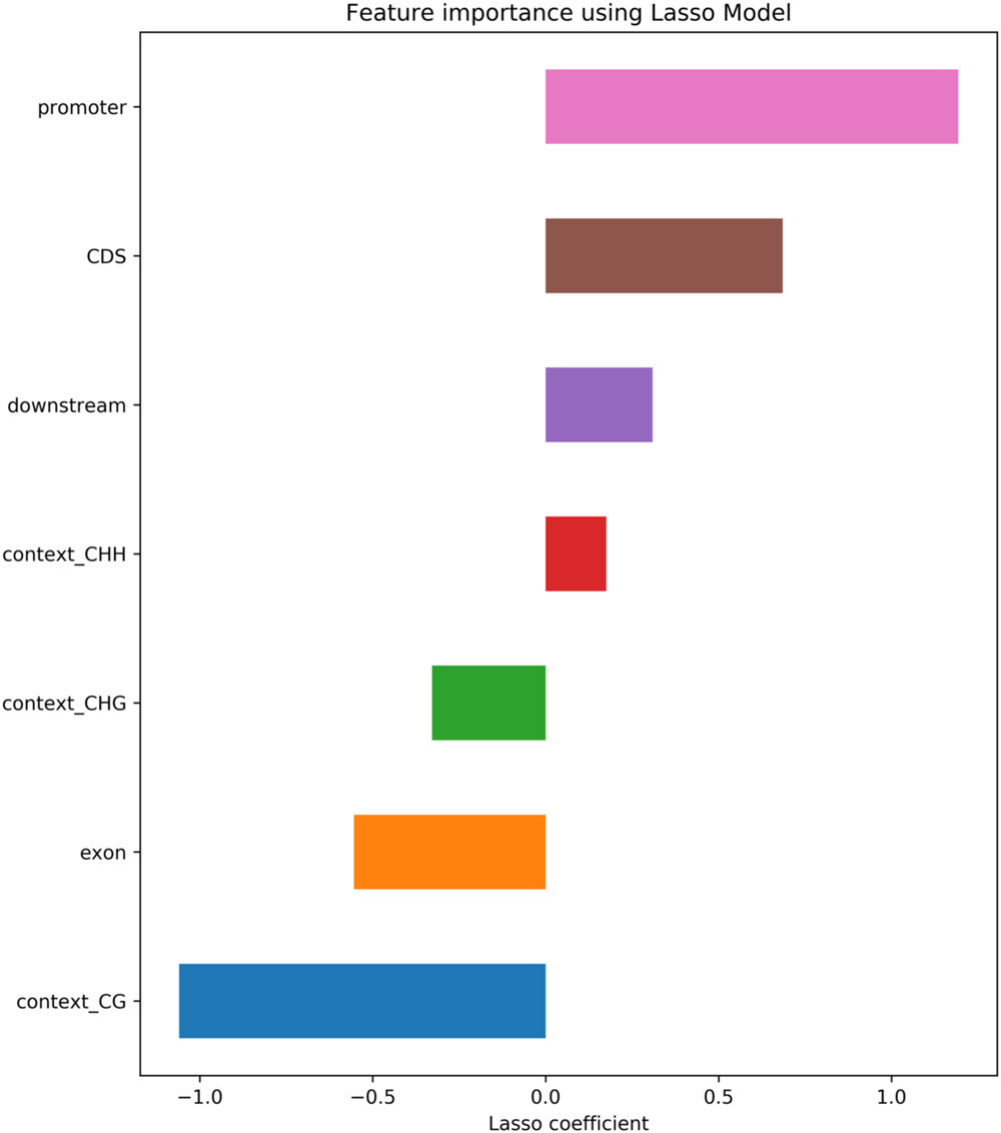
Features importance using Lasso model. The coefficient of the least important features is shrunk toward zero

Both RFE and LASSO did not remove any feature from the matrix of features, suggesting that all of the features contributed to the predictive models. However, the DNA methylation events occurring in CG context, the promoter, the CDS and the exon were greatest (Figure 6).

**FIGURE 7:**
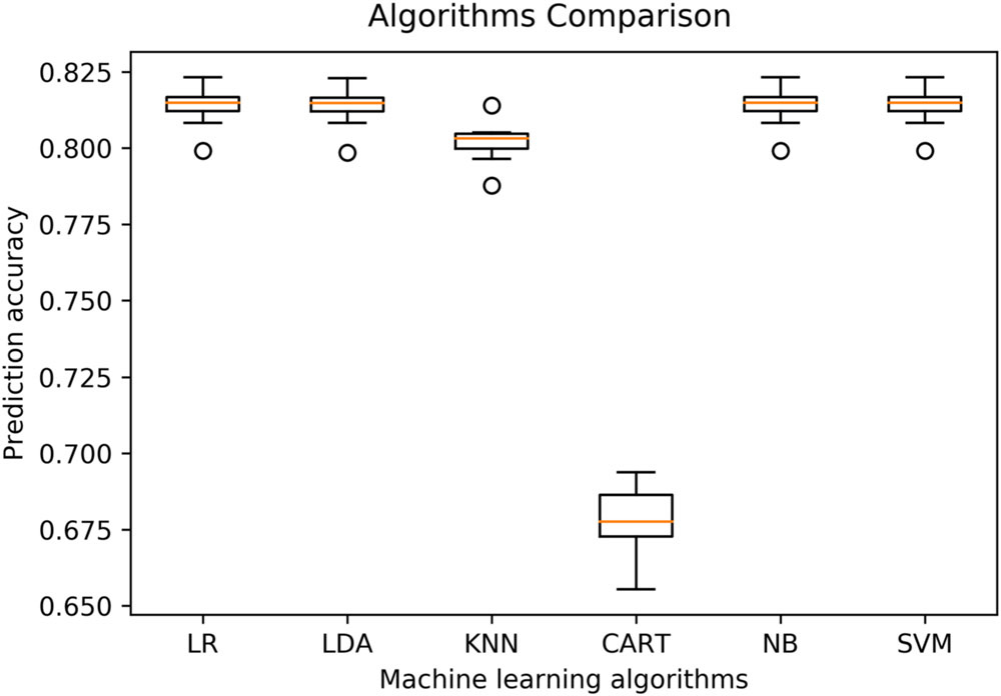
Comparison of prediction accuracies between various machine learning algorithms, Logistic regression (LR), Linear discriminant analysis (LDA), K‐nearest neighbors (KNN), Classification and regression trees (CART), Naïve Bayes (NB) and Support vector machine (SVM)

All of the machine learning algorithms gave on average relatively high prediction accuracies (0.81) of gene expression classes, but CART (0.68) (Figure 7). There was only very little variation around the average prediction accuracies, suggesting that the models consistently predicted the gene expression classes. The Ensemble technique combining the six predictive models and the artificial neural network resulted in similar prediction accuracy (0.81) (Supplemental Figure S5).

## 4 DISCUSSION

In this study, we investigated the DNA methylation patterns across the Chinese Spring genome and their impact on genes expression, contrasting profiles observed between leaf and root tissues. In particular, the methylation levels across annotated genic regions and sequence contexts were used as features to accurately predict differentially expressed genes between roots and leaves, using machine learning and artificial intelligence algorithms.

### 4.1 Identification and distribution of differentially methylated regions (DMRs)

To identify DMRs, a common practice is to segment the genome into equally spaced regions and identify which regions have statistically significant differences in methylation (Bock, 2012; Korthauer, Chakraborty, Benjamini, & Irizarry, 2018; Stockwell, Chatterjee, Rodger, & Morison, 2014). In particular 100‐bp tiles were used for many crops, including rice (Garg, Narayana Chevala, Shankar, & Jain, 2015) and maize (Li et al., 2015; West et al., 2014) whereas a 200‐kb window was used in barley (Chen et al., 2015), a 50‐bp sliding window for *Medicago truncula* (Yaish, Al‐Lawati, Al‐Harrasi, & Patankar, 2018) and 10 kb bins size in Lotus (Lin et al., 2019). However, depending on the bins size, a tile could include only one feature (best case scenario) or a mixture of features. For the sake of demonstration, we randomly picked up an annotated region and split it in bins of 25 kb (Supplemental Figure S6). In this example, the first bin (Bin 1) includes only promoters whereas Bin 2 and Bin 3 include exons and introns. A methylation level for these bins would confound the methylation patterns of the exon and intron. To overcome this issue, we defined genic regions using the annotation of the Chinese Spring genome and we computed the weighted methylation levels for each genic region (Schultz et al., 2012). The benefit of this technique would be the ability to uncover the methylation levels of each specific feature and eventually, relate them to gene expression. Similar approaches have been applied for methylation profiles for other species, such as Lotus (Lin et al., 2019), poplar (Ding, Liang, Diao, Su, & Zhang, 2018) and Brassica (Liu, Xia, Liu, Dai, & Hou, 2018). Nonetheless, most of the authors only consider the flanking regions and the gene body as a whole (Bewick & Schmitz, 2017; Liu et al., 2018; Zilberman, 2017).

Throughout the Chinese Spring genome and regardless of the organ (roots, leaves), ∼60, ∼35, and ∼5% of cytosine residues were methylated in CG, CHG and CHH context, respectively. These genome‐wide methylation patterns are consistent with results of other plant species where the majority of DNA methylation occurs in the CG context (Law & Jacobsen, 2010). In particular, (Gardiner et al., 2015) reported that in non‐transcribed regions of Chinese Spring genome, 59, 35, and 6% of methylated cytosines occurred in CG, CHG and CHH context. In soybean, 60% CG was methylated while only 9% CHH was (An et al., 2017). The percentages of methylcytosines found at CG, CHG and CHH sites in rice were 54.7, 37.3, and 12.0%, respectively (Li et al., 2012). However, making a direct comparison of methylation patterns between different genomes is not trivial because genomes are dynamic and vary in size (Bennetzen & Wang, 2014; Bennetzen, Ma, & Devos, 2005), ploidy (Adams & Wendel, 2005), gene content (Swanson‐Wagner et al., 2010) and repetitive elements (Ausin et al., 2016; Bennetzen & Wang, 2014). DNA methylation is affected by all of these factors and varies substantially within species (Dubin et al., 2015; Eichten et al., 2013; Schmitz et al., 2013; Takuno, Ran, & Gaut, 2016), between different genomic and genic regions, as well as different plant tissues and organs (Chwialkowska, Nowakowska, Mroziewicz, Szarejko, & Kwasniewski, 2016; Roessler, Takuno, & Gaut, 2016).

Because the gene transcript is synthetized using one of the two DNA strands as template, we examined whether DNA methylation occur preponderantly on a specific strand. The two DNA strands showed similar methylation patterns. This result is in agreement with studies in other plants, such as soybean (Song et al., 2013). However, asymmetries in DNA methylation were reported in carrot (Zhou, Magill, Magill, & Newton, 1998) and *Arabidopsis* (Luo & Preuss, 2003).

The methylation levels varied among genic regions (Table 1) and the promoter showed the highest methylation ratio in all contexts. Our result is consistent with other species, including rice (Zhang et al., 2018a), chickpea (Bhatia, Khemka, Jain, & Garg, 2018b), lotus (Lin et al., 2019) and black poplar (Ding et al., 2018) where promoter regions were the most heavily methylated as compared to the other genic regions. In contrast, promoter regions are barely methylated in *Arabidopsis* (Zhang et al., 2006). Promoter‐methylated genes were reported to have tissue‐specific expression (Zhang et al., 2006) and was often associated with transcriptional repression (Li et al., 2008). However, a recent study conducted on apples did not find any correlation between promoter methylation and gene expression (Xu et al., 2018).

The methylation levels in CDS and exons were similar and lower than the promoter regions in all sequence contexts, which is in accordance with findings in apple (Xu et al., 2018) and black poplar (Ding et al., 2018). In our study, when retrieving genic regions from the annotated genome of Chinese spring, many genes were missing 5′‐UTR and/or 3′‐UTR region. Therefore, UTRs were discarded from the analyses. However, some studies reported that 5′‐UTR region in certain genes were more prone to undergo epigenetic modifications compared to promoters (Jiménez‐Garza, Reynaga‐Ornelas, & Dávalos‐Pérez, 2016) and UTRs modulate gene expression and protein function (Srivastava, Lu, Zinta, Lang, & Zhu, 2018). Therefore, when more comprehensive annotation of the wheat genome becomes available, 5′‐UTR and 3′‐UTR methylation levels should be considered in order to get a broader picture of methylation patterns and their relationship with genes expression.

### 4.2 Relationships between methylation patterns and gene expression

To further decipher the relationship of methylcytosines with gene expression, we used DNA methylation levels across annotated genic regions as features to predict differentially expressed genes between root and leaf tissues. Both the recursive feature elimination and LASSO algorithms did not remove any feature, suggesting that methylation levels at all genic regions (promoter, CDS, exon and downstream region) contributed to the predictive model, regardless of the sequence context (CG, CHG and CHH). However, the DNA methylation events occurring in CG context, the promoter, the CDS and the exon showed the highest contribution to the models (Figure 7). This result is congruent with the two‐way analysis of variance that revealed significant (p‐value = 9.20×10^−77^) correlation between all features and gene expression classes (Table 3).

The involvement of DNA methylation in diverse biological processes is well known, including transposon silencing (Sigman & Slotkin, 2016), regulation of gene expression (Zilberman, Gehring, Tran, Ballinger, & Henikoff, 2006), heterosis (Groszmann et al., 2011) and genomic imprinting (Rodrigues & Zilberman, 2015). However, the interplay between methylation patterns and gene expression remains complex. In this study, we computed the weighted methylation levels (Schultz et al., 2012) for each genic region (promoter, CDS, exons, introns and downstream region). Overall, there were significant (p‐value < .001) positive correlations between all features and gene expression, except methylation in CDS and exon that were negatively correlated with gene expression in CHG context (Table 2). These results are congruent with several studies which reported that DNA methylation levels are different among genomic regions and are correlated with gene expression (Brenet et al., 2011; Lin et al., 2019; Wang et al., 2015; Yang et al., 2015). For example, DNA methylation in CG context within the promoter inhibits gene expression (Bell & Felsenfeld, 2000), but the same methylation pattern within exons enhances gene expression (Bell & Felsenfeld, 2000; Cokus et al., 2008). In particular, positive correlations between expression levels and DNA methylation levels in the gene body were reported in rice (Li et al., 2012) and poplar (Liang et al., 2019). Positive correlation of DNA methylation with gene expression has been suggested to be either a cause or consequence of transcription (Rountree & Selker, 1997). However, many studies showed that correlations between methylome and transcriptome varied according to multiple factors, including the sequence context, the methylated region within the gene, and environmental conditions (Chuang, Chen, & Chen, 2012; Flores et al., 2013; Liang et al., 2019; Wang et al., 2015).

Our study clearly demonstrated that using DNA methylation patterns as features, ML and AI combined with gene expression data could be used to provide insight into the functional role of DNA methylation on DGE. Because all of the predictive models, except CART, gave similar accuracy in predicting gene expression classes, any of them could be deployed for practical use. Since epigenetic variations can contribute to phenotypic plasticity and phenotype persistence (Bräutigam et al., 2013; González‐Recio, 2012), methylation data are being used for predicting various traits, such as plant height in *Arabidopsis* (Hu, Morota, Rosa, & Gianola, 2015) and more complex traits including flowering time and primary root length (Cortijo et al., 2014). However, it is noteworthy that other epigenetic mechanisms, such as histone modifications, can also regulate gene expression (Dong & Weng, 2013; McCarthy, 2013; Stillman, 2018). Depending on modification types (e.g., methylation or acetylation) and targets (e.g., which amino acids are at the histone tail), histone modifications can either enhance or repress expression (Cheung & Lau, 2005). The advent of third‐generation sequencing methods, such as Pacific Bioscience's single‐molecule real‐time (SMRT) sequencing (Rhoads & Au, 2015), and Oxford Nanopore Technologies’ nanopore sequencing (https://nanoporetech.com/), might provide a complete picture of the epitranscriptome landscape (Helm & Motorin, 2017; Zhao et al., 2019). When the cost of these new technologies for quantifying the array of epigenetic modifications becomes less prohibitive, we suggest combining these epigenetic marks with DNA methylation data to better dissect the impact of the epigenome on gene expression.

The GO enrichment analysis clearly separated the three gene expression classes according to molecular functions, biological processes and cellular components, corroborating the involvement of DNA methylation in regulating genes associated with these processes between leaves and roots. In particular, genes upregulated in roots were involved in movement of cells or subcellular components and DNA packaging, whereas downregulated genes were mainly involved photosynthesis and pigment biosynthesis processes. Genes that were not differentially expressed between roots and leaves were mainly involved in protein processes (e.g., amino acid biosynthesis and protein transport) and membrane structures. The importance of methylation in various biological processes is well known (Deleris, Halter, & Navarro, 2016; Liang et al., 2019; Zhang et al., 2018b). Because DNA methylation can lead to heritable phenotypic changes (Kinoshita & Seki, 2014; Sano, Kamada, Youssefian, Katsumi, & Wabiko, 1990; Thiebaut et al., 2019), it would be valuable to use methylome data to predict phenotype variations under different environmental conditions, including biotic and abiotic stresses.

However, it is worth mentioning that the tissue‐specific pattern of gene regulation goes beyond the variations in methylation profiles. While tissue specificity is often associated with gene expression levels, it is more likely that individual genes, or even sets of genes, cannot fully capture the variety of processes that distinguish different tissues. Rather, biological function requires the involvement of multiple regulatory elements, primarily transcription factors, which work together with other genetic and environmental factors to mediate the transcription of genes and their protein products (Huang et al., 2018; Vaquerizas, Kummerfeld, Teichmann, & Luscombe, 2009). Thus, although GO analyses give some clues about biological processes, an integrative approach including gene regulatory networks controlling specific traits or different developmental stages would allow uncovering directly the genes involved in these processes. Because organ‐specific gene expression helps understanding the fundamental questions of development as well as identifying promoters’ specific to a single organ, a broader study including various vegetative and reproductive organs would be needed to validate the specificity of gene regulation patterns.

## 5 CONCLUSION

Our study clearly demonstrated that DNA methylation across genic regions can be used for accurately (accuracy = 0.81) identifying tissue‐specific gene regulation patterns. There was strong correlations between all features and gene expression, suggesting that methylation levels at all genic regions contributed to the predictive models. As anticipated, the methylation of the promoter, the CDS and the exon sequences in the CG context had the biggest effect on gene expression. Combined with new long‐read sequencing technologies, our approach may help better decipher the interplay between the epigenetic marks and the expressome.

## Supporting information

SUPPORTING MATERIAL

SUPPORTING MATERIAL
