# Supplementary material for: Machine learning analyses of methylation profiles uncovers tissue‐specific gene expression patterns in wheat
Source: Plant Genome. 2020 Jun 3;13(2):e20027. doi: 10.1002/tpg2.20027 (PMC12806920; doi:10.1002/tpg2.20027)
Supplement: Supplementary file 2 [file TPG2-13-e20027-s002.docx]

**Fig. S1** Distribution of methylated cytosines in CG, CHG and CHH contexts among chromosomes in roots (**A**) and leaves (**B**).

**Fig. S2** Comparison of methylation levels between chromosome strands across Chinese Spring genome in roots (**A**) and leaves (**B**).

**Fig. S3** Features importance using the complete genes set (genes having all the annotated features, including intron) as estimated by the Lasso model. The coefficient of the least important features is shrunk toward zero.

**Fig. S4** Histograms of gene expression in root (red) and leaf (green). The vertical red and green dash lines represent the 95^th^ percentiles of the distributions in root and leaf, respectively. The counts include all transcripts of the genes.

**Fig. S5** Snapshot of the output of the deep neural networks for predicting gene expression classes, using methylation patterns as features.

**Fig. S6** Example of 25-kb binning approach showing potential mixture of different features. Bin 1 only includes the promoters of the genes whereas Bin 2 and Bin 3 include exons and introns.
